# Association of specific frequency bands of functional MRI signal oscillations with motor symptoms and depression in Parkinson’s disease

**DOI:** 10.1038/srep16376

**Published:** 2015-11-17

**Authors:** Xiaopeng Song, Xiao Hu, Shuqin Zhou, Yuanyuan Xu, Yi Zhang, Yonggui Yuan, Yijun Liu, Huaiqiu Zhu, Weiguo Liu, Jia-Hong Gao

**Affiliations:** 1 Department of Biomedical Engineering, College of Engineering, Peking University, Beijing, 100871, China; 2 Department of Neurology, Brain Hospital Affiliated to Nanjing Medical University, Nanjing 210029, China; 3 School of Life Science and Technology, Xidian University, Xi’an, Shanxi 710071, China; 4 Department of Psychiatry and Psychosomatics, Affiliated ZhongDa Hospital of Southeast University, Institute of Neuropsychiatry of Southeast University, Nanjing 210009, China; 5 Center for MRI Research, Beijing City Key Lab for Medical Physics and Engineering, McGovern Institution for Brain Research, Peking University, Beijing, 100871, China

## Abstract

A novel empirical mode decomposition method was adopted to investigate the dissociative or interactive neural impact of depression and motor impairments in Parkinson’s disease (PD). Resting-state fMRI data of 59 PD subjects were first decomposed into characteristic frequency bands, and the main effects of motor severity and depression and their interaction on the energy of blood-oxygen-level-dependent signal oscillation in specific frequency bands were then evaluated. The results show that the severity of motor symptoms is negatively correlated with the energy in the frequency band of 0.10–0.25 Hz in the bilateral thalamus, but positively correlated with 0.01–0.027 Hz band energy in the bilateral postcentral gyrus. The severity of depression, on the other hand, is positively correlated with the energy of 0.10–0.25 Hz but negatively with 0.01–0.027 Hz in the bilateral subgenual gyrus. Notably, the interaction between motor and depressive symptoms is negatively correlated with the energy of 0.10–0.25 Hz in the substantia nigra, hippocampus, inferior orbitofrontal cortex, and temporoparietal junction, but positively correlated with 0.02–0.05 Hz in the same regions. These findings indicate unique associations of fMRI band signals with motor and depressive symptoms in PD in specific brain regions, which may underscore the neural impact of the comorbidity and the differentiation between the two PD-related disorders.

Depression is commonly seen in Parkinson’s disease (PD)^1^,^2^. The neural correlates of motor disorder and depression in PD are complex and not fully delineated. Depression may not progress linearly with motor severity: it may occur in the early or late stages of the disease, or even before any motor impairment is present^1^,^2^. Studies have indicated a complex relationship between depression and motor defect in PD, that these two aspects may influence separate as well as overlapping neural circuits^3^,^4^,^5^.

Neuroimaging data has revealed that distinct and separable brain activity patterns were linked to depression or motor symptoms in PD^3^. Whereas motor disorders are associated with structural deficits in the substantia nigra, thalamus and striatum structure, depression is associated with problems in the limbic system and basal forebrain^6^,^7^. Currently, little is known about the neural bases of the interaction between or comorbidity of motor and depressive disorders. Besides structural impairment, functional deficits have been observed in the subgenual cortex and orbitofrontal cortex in depressed PD patients^3^. Such defects have also been found in similar brain regions in patients with major depression. Thus, it remains unclear whether the functional changes are general hallmarks of depressive symptoms independent of the motor disorder, or specific to the comorbidity of depression and movement deficiency^8^.

This study was designed to identify brain regions separately impacted by the movement disorder, by depression, as well as by a combined force from both symptoms in PD. As such, we sought to explore the neural networks associated solely with depression or the motor disorder and with the comorbidity of both. We hypothesized that the neural impact of dissociation and interaction of motor and depressive symptoms may involve both the frequency and spatial domains, i.e., the motor and depressive symptoms might separately and interactively alter fMRI signal oscillations in multiple brain areas in connection with multiple frequency bands. Motor impairments in PD are shown to be associated with a generalized slowing of the EEG and MEG frequencies^9^,^10^ and increased beta frequency waves^10^,^11^. But non-motor symptoms such as depression^12^ and dementia^13^,^14^ are related to changes in the alpha and delta rhythms^10^. It is still unclear whether the motor and non-motor symptoms have different representations in different rhythms of the blood-oxygen-level-dependent (BOLD) fMRI signal oscillations.

Multiple ongoing neural processes may co-exist in the same brain area, which are characterized by brain waves of different frequencies^15^. These processes can be reflected by BOLD oscillations in different frequency bands^16^ and may be related to assorted brain functions, such as motor controls and mood regulations. Since BOLD oscillations of different frequency bands may have different physiological implications, these rhythms may be affected by or have different sensitivity towards distinct symptoms in PD. Previous fMRI studies demonstrated that effective treatments for PD such as acute levodopa administration and deep brain stimulations (DBS) alter resting-state BOLD fluctuations in selective frequency bands in the sensorimotor network and mood regulation areas^17^,^18^. With respect to the objective of this investigation, little is actually known about the frequency bands of BOLD oscillations or the corresponding brain areas which are specifically related to motor deficiency, depression, or the interaction of the two aspects at the baseline condition.

Recently, several studies have successfully applied a novel data-driven method, Empirical Mode Decomposition (EMD), to the analysis of the spectral characteristics of BOLD oscillations^19^,^20^. The EMD method automatically isolates the underlying processes of BOLD activities in a data-driven manner and divides the whole frequency band into adaptively determined sub bands without the assumption of linearity, stationarity, or recourse to any rigid priori chosen band-pass filter^19^. The EMD method decomposes the original BOLD signal of each voxel into a finite set of intrinsic oscillatory components, termed intrinsic mode functions (IMFs). Each IMF occupies a unique frequency range: the first IMF (IMF1) occupies the highest frequencies, and the last IMF (IMF5) occupies the lowest, with the other IMFs (IMF2 through IMF4 as appropriate) in between.

In the current study, we employed EMD to decompose the BOLD-fMRI data into different frequency bands (IMFs). The energy of BOLD oscillations contained in each frequency band (IMF) was estimated and its correlations with motor severity, depression, and the interaction between the two were evaluated with a multivariate linear regression model.

## Results

The fMRI data of 59 PD patients were analyzed. Twenty of these patients were diagnosed with depressed Parkinson’s Disease (DPD) according to the Diagnostic and Statistical Manual of Mental Disorders, Fifth Edition (DSM-5), while the other 39 patients were considered having non-depressed Parkinson’s Disease (NDPD) (Table 1). HDRS scores were significantly higher in patients with DPD than those with NDPD (two-sample t-test, *t* = 13.5, *p* < 10^−19^). There was no significant difference in age (two-sample t-test, *t* = 1.27, *p* = 0.21), years of education (two-sample t-test, t = 0.129, p = 0.90), gender (Wilcoxon rank sum test, *z* = 1.58, *p* = 0.11), disease duration (two-sample t-test, *t* = 1.248, *p* = 0.22), UPDRS (two-sample t-test, *t* = 0.138, *p* = 0.90), H&Y (chi-square test, *χ*^*2*^ = 3.746, df = 5, *p* = 0.59) or LED (two-sample t-test, *t* = 0.245, *p* = 0.81) between the NDPD and DPD patients, as shown in Table 1. HDRS was not significantly correlated with UPDRS (*R* = −0.15, *p* = 0.26).

**Figure 1:**
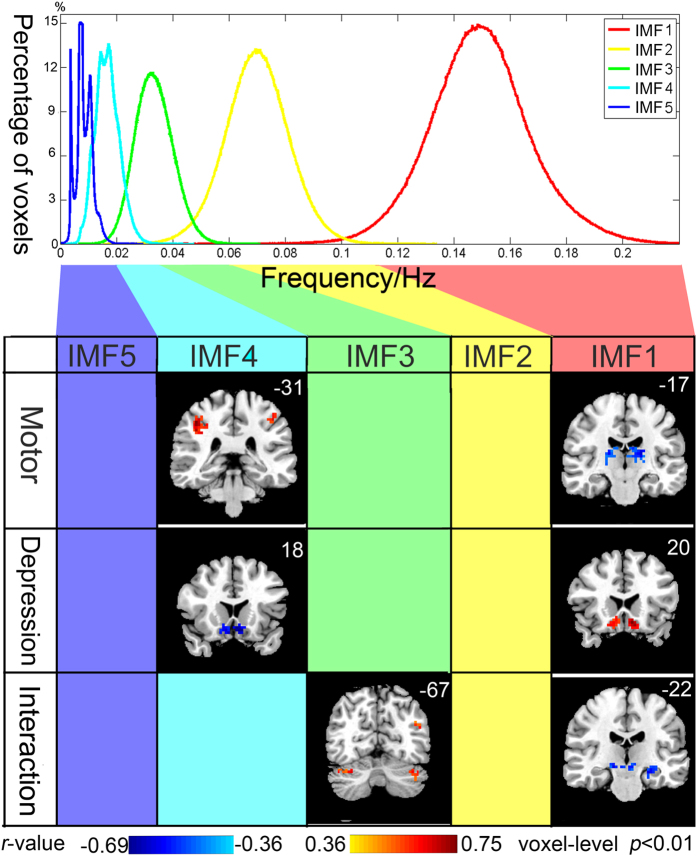
Main effects and interaction of motor and depressive symptoms on the energy of BOLD oscillations in different frequency bands. Upper panel shows the Frequency properties of IMF. EMD yielded five IMFs for each voxel, the mean frequency of each IMF of each voxel was calculated (See supplementary materials for the calculation of the mean frequency of each IMF). Each of the five histograms is a statistics of the whole-brain voxels of all the subjects. Heights of the histograms represent the percentages of voxels whose mean frequency equal to the value on the horizontal axis. Lower panel shows that the main effects of the UPDRS and HDRS were both significant in the bands of IMF1and IMF4, while the interactive effects of UPDRS and HDRS were significant in the bands of IMF1 and IMF3. The blank columns indicate the frequency bands in which the main effects and interactive effects of the UPDRS and HDRS were not significant. Left side of the brain is displayed on the right, Y-MNI coordinate is shown on the upper right corner of each corresponding coronal view.

EMD yielded five IMFs for each voxel. Each IMF occupied a unique frequency band with very slight overlaps among each other (Fig. 1, upper panel): the mean frequency of the IMF1 occupied the highest frequency band (0.1–0.25 Hz); the mean frequency of IMF2 of all the voxels ranged from 0.04 to 0.1 Hz; IMF3 was from 0.02 to 0.05 Hz; IMF4 was from 0.01 to 0.03 Hz; and IMF5 occupied the lowest frequency band from 0 to 0.018 Hz.

Voxel-wise multivariate regression analysis identified the main effects of UPDRS and HDRS scores and their interactions on the energy of BOLD oscillations in various frequency bands (Fig. 1 and Supplementary Table 1, AlphaSim correction, cluster-level P < 0.01, voxel-level *p* < 0.01, cluster size > 28 voxels). Greater motor impairment was associated with lower energy of IMF1 (0.1–0.25 Hz) in the bilateral ventral lateral thalamus, but with higher energy of IMF4 (0.01–0.03 Hz) in the bilateral postcentral gyrus. More severe depressive symptoms were associated with higher energy of IMF1 but lower energy of IMF4 in the bilateral subgenual cingulate. The interaction between motor and depressive symptoms was negatively correlated with the energy of IMF1 in the substantia nigra/ventral tegmental area, left hippocampus, left inferior orbitofrontal cortex, and left temporoparietal junction, but positively correlated with the energy of IMF3 (0.02–0.05 Hz) in left inferior orbitofrontal cortex, left temporoparietal junction, left inferior temporal gyrus, and bilateral cerebellum.

Supplementary Figs 1–3 show the slice views of the main effects of UPDRS, HDRS, and their interaction effects, respectively, with a lower threshold than that in the Fig. 1 (AlphaSim correction, cluster-level P < 0.05, voxel-level *p* < 0.05, cluster size > 54 voxels). The results remained similar to that obtained with the stringent threshold, except for a few additional clusters: IMF1 energy in the cerebellar vermis was negatively correlated with UPDRS; IMF3 energy in the bilateral precentral gyrus, superior and inferior parietal lobule, and middle occipital gyrus were positively correlated with UPDRS.

To further rule out the possibility of head-motion artifacts, we excluded 15 subjects (14 NDPD patients and one DPD patient) whose head motion exceeded 1.0 mm of translation or 1.0° of rotation and repeated the same data analysis procedure on the remaining 45 subjects. The results are shown in Supplementary Figs 4–6. With a stricter head motion exclusion criterion and the same stringent threshold (AlphaSim correction, cluster-level P < 0.01, voxel-level *p* < 0.01, cluster size > 28 voxels), the patterns remained the same as the previous results with 59 subjects.

## Discussion

In the current study, we did not find a correlation between UPDRS and HDRS scores (*r* = −0.1487, *p* = 0.2611), which indicates a non-linear or non-additive relationship between motor and depressive symptoms. Electrophysiological data have suggested that the oscillatory activities across multiple frequency bands are distributed among spatially segregated loops of the basal ganglia-thalamo-cortical system and that these oscillations may relate distinctly to distinct motor or non-motor components of clinical impairments in PD^10^. By applying an innovative data-driven methodology of combining the resting-state fMRI with EMD, we show here for the first time, the neural impact of the dissociation and interaction of motor and depressive symptoms in both the spectral and spatial domains.

We found that the severity of motor symptoms was correlated with decreased energy of IMF1 in the bilateral ventral lateral thalamus in all PD patients. BOLD oscillations of the healthy brains typically revealed relatively higher frequency in the thalamus than in the cortical areas for the maintenance of transmodal functions^21^. We speculated that one of the main effects of motor symptoms might be the decrease of high-frequency power in the ventral lateral thalamus. BOLD oscillations in the thalamus act like a pace maker to the brain. The slowing of these oscillations is consistent with the comprehensive slowing down of background oscillatory activities in cortical EEG and MEG in PD^9^,^10^. This phenomenon may predict motor deficiency in PD.

The energy of IMF4 was positively correlated with UPDRS in the postcentral gyrus, precentral gyrus, superior and inferior parietal lobule, and middle occipital gyrus. These parieto-occipital association regions resemble the dorsal parietal network—one of the several canonical resting-state networks robustly identified by the Independent Component Analysis^22^. This network functions importantly to control visuospatial cued movements and guide spatial tracking^22^. An over-activity in the parietal areas in PD is noted during sequential and bimanual movements, suggesting that there might be a compensatory mechanism for the deficiency in striatal-thalamo-cortical circuits^23^. Consistently with this observation, we have detected the association between motor symptoms and the increased low-frequency oscillation energy (0.01–0.03 Hz) in the parieto-occipital areas, which suggests a possible compensation for motor deficits by invoking sensory and intentional guidance.

The shift of energy from a lower frequency band (IMF4) to a higher frequency band (IMF1) in the subgenual cingulate was discovered to be correlated with depressive symptoms. The subgenual cingulate plays a main role in major depression and has been the target of DBS for the treatment of depression^8^,^24^. According to other resting-state fMRI studies, the amplitude of low frequency fluctuations of BOLD signal in the subgenual cingulate reliably predicts depressive symptoms in PD^3^.

Our results showed that the motor and depressive symptoms might interactively affect the substantia nigra/ventral tegmental area, hippocampus, inferior orbitofrontal cortex, and cerebellum. These results are in agreement with a model of neurodegeneration proposed by Mayberg *et al.* that unified depression and PD^1^,^25^. This model suggests that the primary degeneration of dopaminergic neurons in the substantia nigra/ventral tegmental area leads to dysfunction of the basal ganglia and orbitofrontal cortex, which secondarily influences serotonergic cell bodies in the dorsal raphe nuclei^1^. Both the dopamine and serotonin pathways impact the subgenual cingulate through efferent projections^8^. Additional circuits affected in DPD patients include the basal ganglia-thalamo-cortical circuit, and the basotemporal limbic circuit linking the orbitofrontal cortex to the amygdala, hippocampus, and inferior temporal gyrus through the uncinate fasciculus^26^.

In Mayberg’s model^26^, regions with known anatomical and functional connections that also show significant metabolic changes following successful anti-depression treatments are grouped into three behavioral compartments: the autonomic, the self-reference, and the sensory-cognitive compartment.

The subgenual cingulate is within the autonomic compartment and might be irrelevant to the sensory-motor symptoms. This notion is consistent with our findings that the changes in the subgenual cingulate are correlated with depressive symptoms but not motor symptoms. DBS targeted to the subgenual cingulate and the nucleus accumbens are believed to be able to alleviate depressive symptoms in some cases of major depression. However, similar DBS treatment at the substantia nigra in PD patients may lead to transient, acute major depression^24^. Thus, whilst the subgenual cingulate appears to be an essential region in depression, the substantia nigra may be related to both depression and movement disorders.

The orbitofrontal cortex and medial prefrontal cortex reside in the self-reference compartment, while the temporoparietal junction, inferior temporal gyrus, and hippocampus belong to the sensory-cognitive compartment. Impaired glucose metabolism in the inferior orbitofrontal cortex has been identified in DPD as compared to the NDPD and healthy controls^27^. Depression in PD seems to be correlated with a reduction in the metabolism in the orbitofrontal cortex, whereas reductions in the temporoparietal junction and temporal lobe are associated with sensory-cognitive dysfunctions in PD^7^,^28^. Decreased brain-derived neurotrophic factor (BDNF) in the substantia nigra and hippocampus has been indicated in the pathophysiology of both PD and depression^29^. It has been reported that BDNF levels were not only diminished in the substantia nigra of PD patients, but also in the hippocampus and serum of depressed individuals. Treatment with antidepressant drugs or electroconvulsive stimulation may increase the production of BDNF in the hippocampus and cortex^1^,^29^. In summary, our results along with the accumulating evidence suggest that these limbic-cortical regions may be involved in the progression of both depression and sensory-cognition guided movement deficits.

The interactive effects of motor and depressive symptoms on the bilateral posterior cerebellum were also observed in this study. Previous neuroimaging studies^30^,^31^,^32^,^33^ have found hyperactivation in the cerebellum in both PD and major depressive disorder (MDD). Several studies have reported hyperactivation in the ipsilateral cerebellum beside the normally activated contralateral cerebellum when PD patients performed strictly unilateral hand movement^30^,^31^, suggesting that hyperactivation in the bilateral cerebellum might be a compensatory mechanism for the defective motor system^31^. In depression, however, the increased resting state cerebellar activity may be an indicator of the disease state^32^. Various resting-state studies have observed altered cerebellar-cerebral functional connectivities in patients with drug-naive depression, treatment-resistant depression, and geriatric depression^32^. Increased activation in the cerebellum was also observed during a rewarded sustained-attention task in adolescents with treatment-naive, first-episode MDD^33^. It is widely known that the cerebellum contributes to cognitive and emotional control besides its role in motor coordination^34^. The intimate afferent and efferent connections to the midbrain and limbic system provide the neuroanatomical foundation for the cerebellar involvement in movement and emotional disorders. Both structural^6^ and functional^28^ abnormalities of the cerebellum are present in movement and emotional disorders in DPD patients. It is probably not coincidental that depression is often associated with psychomotor disturbances involving gait, posture, and coordination of movements, which are typically found in cerebellar ataxia^34^.

In conclusion, our novel findings demonstrate unique associations of fMRI band signals with motor and depressive symptoms in PD in specific brain regions. These data may provide a neural basis for the dissociative and interactive relations between the two PD-related disorders and have obvious clinical implications. Since the pathogenesis of depression in PD differs considerably from depressed patients without PD, the commonly used treatments for depression may be ineffective in ameliorating depressive symptoms in PD^35^. The current investigation constitutes precisely the kind of research needed to delineate the neural interactions as well as dissociations underlying motor and depression symptoms in PD and in turn offers guidance for developing interventions targeting at specific components of clinical impairments in Parkinson’s disease.

## Materials and Methods

### Participants

The study was approved by the Medical Research Ethical Committee of Nanjing Brain Hospital in accordance with the Declaration of Helsinki. Sixty patients with idiopathic Parkinson’s disease were recruited (See supplementary materials for the inclusion and exclusion criteria). Written informed consents of all subjects were obtained. We used the Hoehn and Yahr (H&Y) staging scale to determine the disease stage, the Unified Parkinson’s Disease Rating Scale motor part III (UPDRS) to measure motor disability, and the Mini-Mental State Examination (MMSE) to assess global cognitive function. The severity of depression was evaluated by the 17-item Hamilton Depression Rating Scale (HDRS). Previous studies showed that a cut-off of 13/14 in HDRS was appropriate to conclude depression in PD^36^,^37^. Statistical analysis on the demographical variables was performed by using the Statistical Package for Social Sciences (SPSS) version 15 for Windows.

### MRI data acquisition and image preprocessing

MRI experiments were performed using a 3 T Siemens Trio MRI scanner. The participants were instructed to rest with eyes closed, keep the head still, and stay awake. Standard image preprocessing analyses were performed in combination with stringent motion artifact correction procedures (See supplementary material for detailed information). One subject was excluded from the analysis due to excessive head motion.

### Empirical mode decomposition

After preprocessing, EMD^38^,^39^ was performed in a voxel-wise fashion to decompose the time series of a certain voxel into a finite set of time courses of the same length as the original signal, termed IMFs^20^. Each IMF occupies a unique frequency range. The number of IMFs were determined by the intrinsic temporal-spectral characteristics of the original time series rather than predefined. For almost all voxels in the brain, the decomposition of the time course yielded five IMFs, denoted as IMF1 to IMF5. We then calculated the energy of each IMF for each voxel and generated five energy maps for each subject, representing the relative energy contained in the five distinct frequency ranges. See Supplementary material for detailed information.

### Correlation of behavioral scores with the energy in different frequency bands

To determine the correlation between behavioral scores and the energy in different frequency bands, a group-level voxel-wise multivariate linear regression analysis^40^ (see below) was employed for the energy maps in each of the of five frequency bands:


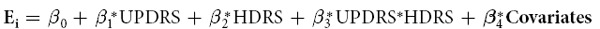


where **E**_**i**_ is the Energy of IMF in a certain voxel of the *i*th subject across the group, *β*_*0*_ is the intercept of the straight line fitting in the model. *β*_*1*_ and *β*_*2*_ are the main effects of UPDRS and HDRS, respectively. *β*_*3*_ represents their interaction on the energy of the voxel. Each element of the vector ***β***_***4***_represents the effect of one of the covariates shown in Table 1, including age, gender, years of education, PD duration, H&Y, levodopa equivalent dose (LED), and MMSE. Group statistic maps of *β*_*1*_, *β*_*2*_, and *β*_*3*_were generated for each of the five frequency bands after multiple comparison corrections. Significant thresholds were set at a corrected cluster-level P value < 0.01 using the AlphaSim program in the AFNI platform (http://afni.nimh.nih.gov, estimated smoothness FWHM = 5.15 mm × 5.07 mm × 4.74 mm, DLH = 1.01, Volume = 70831, RESELS = 4.58, cluster-level P < 0.01, voxel-level p < 0.01, cluster size > 28 voxels). SPM8 (http://www.fil.ion.ucl.ac.uk/spm) was used for the above statistical analysis.

## Additional Information

**How to cite this article**: Song, X. *et al.* Association of specific frequency bands of functional MRI signal oscillations with motor symptoms and depression in Parkinson's disease. *Sci. Rep.* **5**, 16376; doi: 10.1038/srep16376 (2015).

## Supplementary Material

Supplementary Information

## Figures and Tables

**Table 1:** Demographic and neuropsychological characteristics of the subjects.

**Groups**	**NDPD (n = 39)**	**DPD (n = 20)**	***p*** **value**
Age (years)	54.69 ± 10.45	58.05 ± 7.72	0.21
Education (years)	11.33 ± 3.77	11.15 ± 3.12	0.90
Gender (M/F)	26/13	9/11	0.11
HDRS	6.82 ± 3.14	20.45 ± 4.58	<10^−19**^
UPDRSIII	28.21 ± 13.17	27.65 ± 13.17	0.90
H&Y	1.72 ± 0.64	1.40 ± 0.60	0.59
LED (day/mg)	474.18 ± 399.39	500.63 ± 423.71	0.81
PD duration time (years)	6.50 ± 3.54	5.35 ± 2.82	0.22

Values are represented as the mean ± SD. For comparisons of the demographics, *p* values were obtained using two-sample t-tests (*p* value for the gender difference was obtained using Wilcoxon rank sum test, *p* value for the H&Y was obtained using chi-square test). **p < 0.001. HDRS = Hamilton Depression Rating Scale; UPDRS III = Unified Parkinson’s Disease Rating Scale motor part III; H&Y = Hoehn and Yahr Staging Scale; LED = levodopa equivalent dose.
