# Supplementary material for: Association of specific frequency bands of functional MRI signal oscillations with motor symptoms and depression in Parkinson’s disease
Source: Sci Rep. 2015 Nov 17;5:16376. doi: 10.1038/srep16376 (PMC4648086; doi:10.1038/srep16376)
Supplement: Supplementary Information [file srep16376-s1.doc]

**Association of specific frequency bands of**

**functional MRI signal oscillations**

**with motor symptoms and depression in Parkinson’s disease**

Xiaopeng Song^1^, Xiao Hu^2^, Shuqin Zhou^1^, Yuanyuan Xu^2^, Yi Zhang^3^,

Yonggui Yuan^4^, Yijun Liu^1^, Huaiqiu Zhu^1^, Weiguo Liu^2*^, and Jia-Hong Gao^1,5*^

**Supplementary materials**

# Materials and methods

## Participants

The study was approved by Medical Research Ethical Committee of Nanjing Brain Hospital in compliance with the Declaration of Helsinki. Written informed consents of all subjects were obtained. Participants were 60 individuals with idiopathic Parkinson’s disease. All of the participants met the UK Parkinson's Disease Society Brain Bank criteria for idiopathic Parkinson’s disease. Exclusion criteria were: (1) Mini-Mental State Examination (MMSE) scores < 25; (2) acute physical illness or other primary neurological illness; (3) a history of brain injury or electroconvulsive therapy; (4) T_2_-weighted MRI showing major WM changes, infarction, or other lesions; (5) use of antidepressants within one year from the beginning of the study. Confirmation of the diagnosis of depression was also made by an experienced clinical psychiatrist according to DSM-V. Analysis for motion artifact occurred prior to any further image processing or analysis. One subject was excluded from the study due to excessive head motion.

## MRI Data Acquisition and image Preprocessing

Imaging was performed using a 3T Siemens Trio MRI scanner at Nanjing Brain Hospital, Nanjing, China. Axial anatomical images were acquired using a T_1_-FLAIR sequence (TR/TE = 2530 ms/3.34 ms, flip angle = 7^◦^, matrix = 256×192, FOV = 256 mm × 256 mm, slice thickness/gap = 1.33 mm/0.5 mm, 128 slices covered the whole brain) for image registration and functional localization. Functional images were subsequently collected in the same slice orientation as the gradient-recalled echo-planar imaging pulse sequence (TR/TE = 2000 ms/30 ms, flip angle = 90^◦^, matrix = 64 × 64, FOV = 220 mm × 220 mm, thickness/gap = 3.5 mm/0.6 mm, in-plane resolution = 3.4 mm × 3.4 mm, slices numbers = 31). The resting-state fMRI scan lasted for 290s, containing 145 brain volumes. One subject with translation or rotation parameters exceeded ±2 mm or ± 2 degree was excluded.

## Image Preprocessing

Images were analyzed using the following procedure with SPM8 (<http://www.fil.ion.ucl.ac.uk/spm>) and DPARSF software (<http://rfmri.org/DPARSF>). The first five time points were removed to eliminate non-equilibrium effects of magnetization. The remaining 140 volumes of functional BOLD images were corrected for slice timing effects, realigned, and corrected for motion artifacts. The linear trend, mean white matter and CSF signals, and the 24 head motion covariates (translations, rotations, their derivatives, and quadratic terms) were regressed out from each voxel’s time course. Functional volumes for each subject were registered to the corresponding high-resolution T_1_-weighted image by using the Diffeomorphic Anatomical Registration Through Exponentiated Lie Algebra (DARTEL), which was then normalized to the standard MNI brain template, resulting in a functional image series of 61 × 73 × 61 voxels (voxel size of 3 mm × 3 mm × 3 mm). Images were then spatially smoothed with 4 mm × 4 mm × 4 mm FWHM DARTEL T_1_ weighted smoothing kernel. The time course of each voxel was then detrended and normalized by subtracting its own temporal mean and divided by its own temporal standard deviation.

## Empirical mode decomposition

Mathematically, for a real-valued BOLD signal$\text{ }x\left(t\right)$, the standard EMD finds a set of 𝐾 IMFs {$IMF_{i}\left(t\right)$}, *i* =1 to *K*, and a monotonic residue signal$r\left(t\right)$, so that

$x\left(t\right)=\sum_{i=1}^{K} IMF_{i}\left(t\right)+r\left(t\right)$ (1)

To ensure that the time frequency spectra yield meaningful frequency estimates (e.g. no negative frequencies), IMFs{$IMF_{i}\left(t\right)$} are defined so as to have symmetric upper and lower envelopes, with the number of zero crossings and the number of extrema differing at most by one. To extract IMFs using EMD, an iterative method known as the sifting algorithm is used. For illustration, a sifting procedure for obtaining the first IMF (IMF1) from the signal is outlined in the algorithm below.

The standard EMD algorithm:

1) Find the locations of all the extrema of$\text{ }x\left(t\right)$;

2) Interpolate between all the minima (resp. maxima) to obtain the lower (resp. upper) signal envelope, $elow\left(t\right)$ (resp.$\text{ }eup\left(t\right)$);

3) Compute the local mean time course;

4) Obtain the “oscillatory mode” from$r\left(t\right)=x\left(t\right)-emean\left(t\right)$;

5) If $r\left(t\right)$ obeys the stopping criteria, $IMF_{i}\left(t\right)=r\left(t\right)$ becomes an IMF, otherwise set $x\left(t\right)=r\left(t\right)$ and repeat the process from Step 1.

To obtain remaining IMFs, the same procedure is applied iteratively to the residual $r\left(t\right)=x\left(t\right)-IMF_{i}\left(t\right)$until we are left with the monotonic signal. The standard stopping criterion terminates the sifting process only after the IMF condition is met for 𝑆 consecutive times (𝑆 is normally taken to be 2 or 3), here 𝑆 = 3.

## Hilbert transform

After the extraction of IMFs from the original signal, say *X(t)* is one of them, using HT, we can perform its Hilbert Transform $Y(t)$ using:

$Y(t)=\frac{1}{\pi}P\int\frac{X(t')}{t-t'}dt$ (2)

where 𝑃 is the Cauchy principle value:


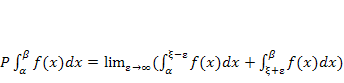
 (3)

Using this definition, both *X*(𝑡) and 𝑌 (𝑡) can be combined to form an analytic signal, 𝑍(𝑡), given by:


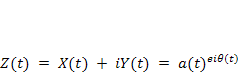
(4)

where 𝑎(𝑡) = [𝑋^2^(𝑡)+𝑌^2^(𝑡)]^1/2^ is the envelope of the signal and 𝜃(𝑡) = tan^−1^(𝑌/𝑋) represents its instantaneous phase. The instantaneous frequency of the Hilbert spectrum can now be defined using:


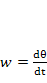
 (5)

By applying the Hilbert transform to individual IMFs, the EMD decomposed original signal
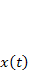
 (omitting the residue) can be expressed as


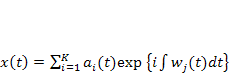
 (6)

## Calculation of HWF

Next, considering the total number of 𝑁 time points of the input signal *x*(𝑡), the Hilbert weighted frequency
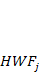
 of each IMF is obtained by taking the weighted mean of instantaneous frequencies
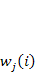
 using:


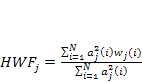
 (7)

The Hilbert weighted frequency (HWF) (Song et al., 2014; Xie and Wang, 2006) of each IMF was determined using instantaneous information about amplitude and phase to reflect the mean oscillation frequency of the IMF. HWF is comparable to the traditional Fourier frequency and is measured by units of Hertz. The HWF reflects the mean frequency of an IMF by summarizing its spectral characteristics. For example, when the HWF of an IMF time course equals 0.15Hz, it indicates that more energy is distributed around 0.15Hz and the Fourier power spectrum of this IMF peaks around 0.15Hz. The discussion of how to calculate HWF is beyond the scope of this work, and readers can refer to the literature for a more detailed description of the calculation (Xie and Wang, 2006).

We then calculated the HWF of IMF1 to IMF5 for each voxel to get the histograms of HWF distribution of IMF1 to IMF5 for the voxels in the whole brain. Our results showed that the frequency of the same IMF (IMF_i_, i=1, 2, 3, 4 or 5) for all the voxels across the brain and subjects were roughly similar and were generally higher than the frequency of the next IMF (IMF_i+1_). The energy of each IMF was defined as the quadratic sum of the IMF time series. Five energy maps were generated for each subject.

The histograms of HWF distributions for the five IMFs of the voxels in the whole brain across all the subjects were shown in Fig. 1. Each of the five histograms is a statistics of the whole-brain voxels of all the subjects. Since the frequency content of different voxels at different sites of the brain (and subjects) are similar, the same IMF (IMF_i_, i=1, 2, 3, 4 or 5) from any voxel will generally fall into the same frequency band. Hence, the Hilbert weighted frequencies of IMF1 of all the voxels in the brains of all the subjects range from 0.01 to 0.25Hz and are generally higher than the frequencies of IMF2; this is the same as the other IMFs. As shown in Fig. 1, each IMF occupies a unique frequency band with very slight overlap: the first IMF (IMF1) occupies the highest frequencies (0.1-0.25Hz); the frequencies of IMF2 for all the voxels range from 0.04 to 0.1Hz; IMF3 frequencies range from 0.02 to 0.05Hz; IMF4 frequencies range from 0.01 to 0.03Hz; and IMF5 occupies the lowest frequency band (0 to 0.02Hz). These frequency properties of IMFs make the energy of the same IMF comparable across different brain areas and different subjects.

**Supplementary Table 1.** Brain regions whose BOLD oscillation energy in specific frequency bands were significantly correlated with UPDRS, HDRS, or the interaction between UPDRS and HDRS.

|  | Frequency band | Brain region | side | BA | cluster size | MNI | R statistic | Cluster P Value |
| --- | --- | --- | --- | --- | --- | --- | --- | --- |
| UPDRS | IMF1 | Thalamus Ventral Lateral Nucleus | L |  | 75 | -21 -21 12 | -0.67514 | 3.143×10^-3^ |
|  |  | Thalamus Ventral Lateral Nucleus | R |  | 47 | 18 -12 12 | -0.56745 | 2.932×10^-3^ |
|  | IMF4 | Postcentral | L | 3,40 | 99 | -42 -24 45 | 0.55337 | 3.832×10^-3^ |
|  |  | Postcentral | R | 3,40 | 60 | 42 -33 45 | 0.57861 | 3.678×10^-3^ |
| HDRS | IMF1 | subgenual cingulate | L,R | 25 | 60 | -12 18 -15 | 0.52664 | 2.135×10^-3^ |
|  | IMF4 | subgenual cingulate | L,R | 25 | 75 | -6 18 -15 | -0.51263 | 1.905×10^-3^ |
| interaction | IMF1 | substantia nigra/ventral tegmental area | L |  | 89 | -6 -20 -8 | -0.47639 | 4.349×10^-3^ |
|  |  | substantia nigra/ventral tegmental area | R |  | 73 | 6 -14 -5 | -0.47319 | 4.267×10^-3^ |
|  |  | Hippocampus | L | 34 | 60 | -2 -12 -18 | -0.52018 | 2.238×10^-3^ |
|  |  | Inferior orbital frontal gyrus | L | 47 | 69 | -39 27 -21 | -0.63825 | 1.852×10^-3^ |
|  |  | Temporal parietal junction | L | 39 | 87 | -48 -66 21 | -0.54864 | 2.013×10^-3^ |
|  | IMF3 | Inferior orbital frontal gyrus | L | 47 | 45 | -42 39 -12 | 0.55335 | 2.116×10^-3^ |
|  |  | Temporal parietal junction | L | 39 | 53 | -48 -78 24 | 0.62432 | 1.878×10^-3^ |
|  |  | Inferior Temporal Gyrus | L | 20 | 82 | -57 -48 -12 | 0.65078 | 1.403×10^-3^ |
|  |  | Cerebellum Posterior Lobe | L |  | 60 | -33 -78 -24 | 0.56858 | 2.483×10^-3^ |
|  |  | Cerebellum Posterior Lobe | R |  | 31 | 30 -60 -33 | 0.61802 | 1.252×10^-3^ |

*Note.* HDRS = Hamilton Depression Rating Scale; UPDRS = Unified Parkinson’s Disease Rating Scale; IMF = Intrinsic Mode Functions; L = left side; R = right side.

# Figure Legends

**Supplementary Figure 1. Main effects of motor symptoms.** Clusters that were positively and negatively correlated with UPDRS were shown in warm and cold colors, respectively (voxel-level p <0.05, cluster size>54 voxels). (A). Brain areas whose BOLD oscillation energy in IMF1 were correlated with UPDRS. (B). Brain areas whose BOLD oscillation energy in IMF4 were correlated with UPDRS. Left side of the brain is displayed on the right.

**Supplementary Figure 2. Main effects of depressive symptoms.** Clusters that were positively and negatively correlated with HDRS were shown in warm and cold colors, respectively (voxel-level p <0.05, cluster size>54 voxels). (A). Brain areas whose BOLD oscillation energy in IMF1 correlated with HDRS. (B). Brain areas whose BOLD oscillation energy in IMF4 correlated with HDRS. Left side of the brain is displayed on the right.

**Supplementary Figure 3. Interactive effects of motor and depressive symptoms.** Clusters that were positively and negatively correlated with the interactive effects of motor and depressive symptoms were shown in warm and cold colors, respectively (voxel-level p <0.05, cluster size>54 voxels). (A). Brain areas whose BOLD oscillation energy in IMF1 were associated with the interaction between UPDRS and HDRS. (B). Brain areas whose BOLD oscillation energy in IMF3 were associated with the interaction between UPDRS and HDRS. Left side of the brain is displayed on the right.

**Supplementary Figure 4. Main effects of motor symptoms calculated with a stricter head motion exclusion criterion.** Clusters that were positively and negatively correlated with UPDRS were shown in warm and cold colors, respectively (AlphaSim correction, cluster-level P < 0.01, voxel-level *p* < 0.01, cluster size > 28 voxels). (A). Brain areas whose BOLD oscillation energy in IMF1 were correlated with UPDRS. (B). Brain areas whose BOLD oscillation energy in IMF4 were correlated with UPDRS. Left side of the brain is displayed on the right.

**Supplementary Figure 5. Main effects of depressive symptoms calculated with a stricter head motion exclusion criterion.** Clusters that were positively and negatively correlated with HDRS were shown in warm and cold colors, respectively (AlphaSim correction, cluster-level P < 0.01, voxel-level *p* < 0.01, cluster size > 28 voxels). (A). Brain areas whose BOLD oscillation energy in IMF1 correlated with HDRS. (B). Brain areas whose BOLD oscillation energy in IMF4 correlated with HDRS. Left side of the brain is displayed on the right.

**Supplementary Figure 6. Interactive effects of motor and depressive symptoms calculated with a stricter head motion exclusion criterion.** Clusters that were positively and negatively correlated with the interactive effects of motor and depressive symptoms were shown in warm and cold colors, respectively (AlphaSim correction, cluster-level P < 0.01, voxel-level *p* < 0.01, cluster size > 28 voxels). (A). Brain areas whose BOLD oscillation energy in IMF1 were associated with the interaction between UPDRS and HDRS. (B). Brain areas whose BOLD oscillation energy in IMF3 were associated with the interaction between UPDRS and HDRS. Left side of the brain is displayed on the right.


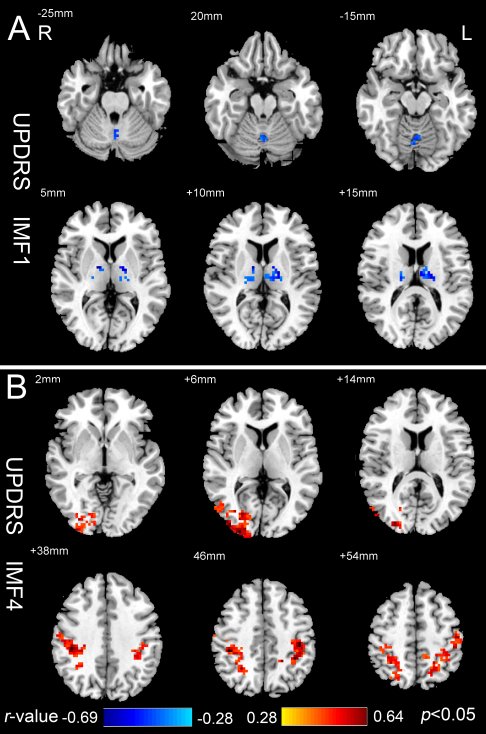


**Supplementary Figure 1. Main effects of motor symptoms.**

**
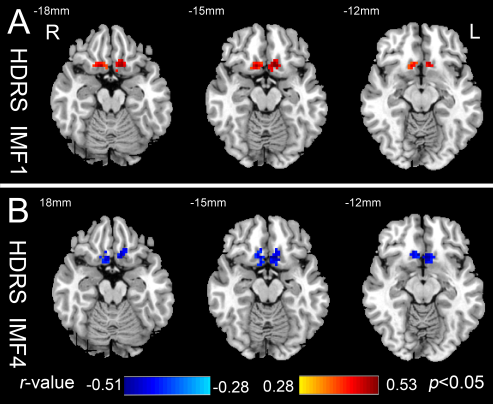
**

**Supplementary Figure 2. Main effects of depressive symptoms.**

**
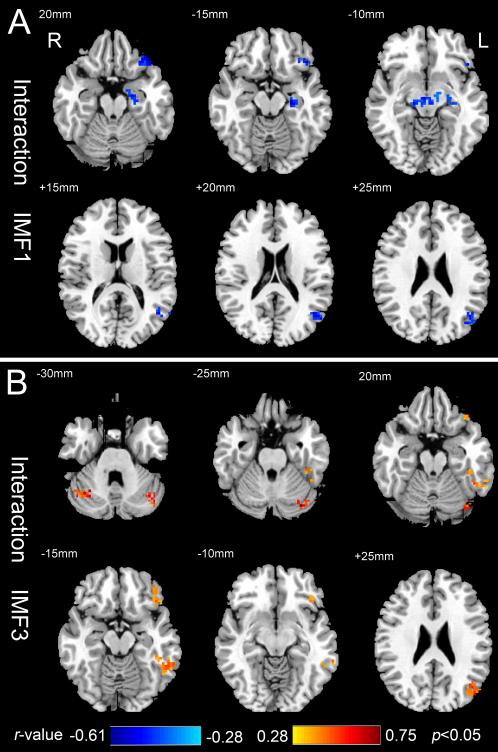
**

**Supplementary Figure 3. Interactive effects of motor and depressive symptoms.**


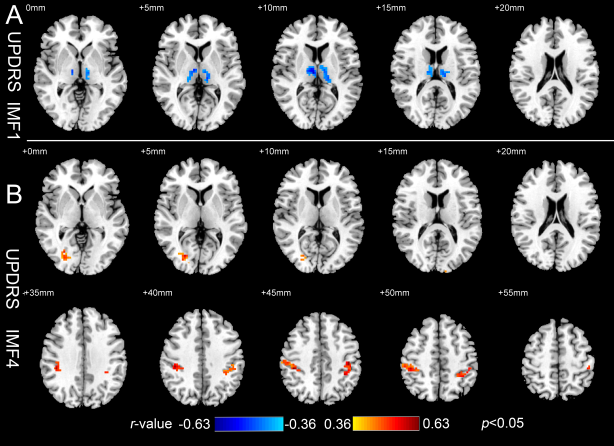


**Supplementary Figure 4. Main effects of motor symptoms calculated with a stricter head motion exclusion criterion.**

**
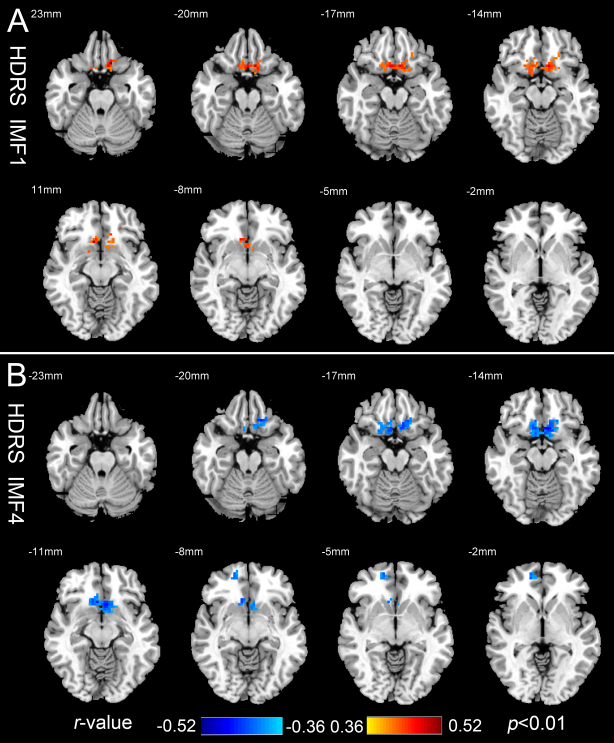
**

**Supplementary Figure 5. Main effects of depressive symptoms calculated with a stricter head motion exclusion criterion.**

**
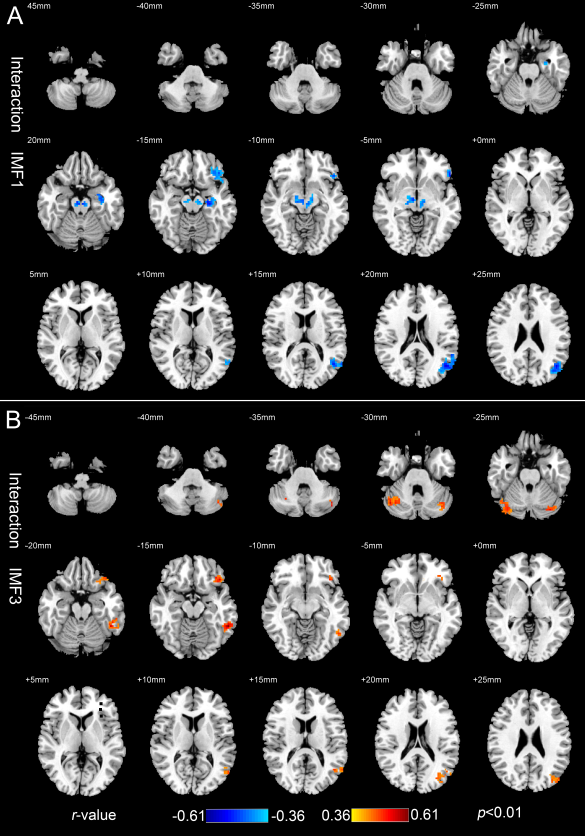
Supplementary Figure 6. Interactive effects of motor and depressive symptoms calculated with a stricter head motion exclusion criterion.**

**References:**

Song X, Zhang Y, Liu Y. Frequency specificity of regional homogeneity in the resting-state human brain. PLOS ONE 2014; 9: e86818.

Xie H, Wang Z. Mean frequency derived via Hilbert-Huang transform with application to fatigue EMG signal analysis. Computer methods and programs in biomedicine 2006; 82: 114-120.
